# Visual Impairment, Hearing Loss and Cognitive Function in an Older Population: Longitudinal Findings from the Blue Mountains Eye Study

**DOI:** 10.1371/journal.pone.0147646

**Published:** 2016-01-25

**Authors:** Thomas Hong, Paul Mitchell, George Burlutsky, Gerald Liew, Jie Jin Wang

**Affiliations:** Centre for vision research, Department of Ophthalmology and Westmead Millennium Institute, University of Sydney, Sydney, Australia; Medical College of Soochow University, CHINA

## Abstract

The presence of visual impairment (VI) and hearing loss (HL) with may be a marker for subsequent cognitive decline over time in older people. A prospective, longitudinal population-based study of the 3654 participants of the Blue Mountains Eye Study were assessed for the associations between VI and HL and a decline in mini-mental state examination (MMSE) scores over a duration of 10 years from the 5-year (baseline of this report) to the 15-year follow-up visits. MMSE was assessed at the 5-, 10- and 15-year follow-up visits. A decline ≥3 scores from 5-year to 10- or 15-year visits indicated possible cognitive decline. VI was defined as best-corrected visual acuity <6/12 in the worse-eye, HL was defined as pure-tone average >40 decibels in the worse-ear and dual sensory impairment (DSI) was defined by the co-presence of VI and HL, detected at 5-year follow-up (baseline of this report). Participants with no VI and HL over the same 5- or 10-year corresponding period were controls. Associations of VI, HL and DSI with possible cognitive decline were assessed using logistic regression models adjusting for age and sex after excluding subjects with a stroke history. The presence of VI, HL or DSI was not associated with possible cognitive decline over 5 years (odds ratio (OR) 0.84, 95% confidence-intervals (CI) 0.40–1.79, OR 1.02, 95% CI 0.61–1.70 and 1.41, 95% CI 0.54–3.72, respectively) or 10 years (OR 1.09, 95% CI 0.52–2.30, OR 1.09, 95% CI 0.65–1.82 and 1.15, 95% CI 0.28–4.73, respectively). There were no changes to these findings after adjustment for other potential confounders. Age was significantly associated with possible cognitive decline (OR 1.07, 95% CI 1.04–1.10 for both periods). Neither visual impairment, hearing loss nor dual sensory impairment was independently associated with subsequent decline in cognition.

## Introduction

Several cross-sectional studies previously reported associations between sensory impairments (the presence of visual impairment, hearing impairment and combined visual and hearing impairment) and cognitive decline[1–3], including a report using Blue Mountains Eye Study (BMES) baseline data[4] that showed a weak but significant cross-sectional association between sensory and cognitive function. However, findings of these associations using samples drawn from community dwelling or institutionalized older residents have been inconsistent after adjusting for potential confounding factors[1–4].

Only a limited number of longitudinal studies have assessed the associations between sensory and cognitive function decline in older persons, and conflicting findings have been reported from the few studies conducted[5–7]. Limitations of previous studies include unclear definitions of sensory impairment (the loss of visual or hearing function), use of non-standardized methods to assess visual and hearing function, and cognitive function assessment tools have included vision-related testing items.

In this report we aimed to determine whether longitudinal associations existed between visual, hearing and dual sensory impairment and possible cognitive decline over 5 and 10 years in a population-based cohort of older Australians, using the mini-mental state examination (MMSE) instrument, after removing visually dependent tasks (termed MMSE blind).

## Methods

### Population

The BMES is a population-based cohort study of vision and common eye diseases in a suburban Australian population 49+ years residing in the Blue Mountains area, west of Sydney. Detailed methods of the baseline examinations were reported previously[8]. Briefly, at baseline the study recruited and examined 3654 participants between 1992 and 1994. Survivors of baseline participants were invited to participate in the 5-, 10- and 15-year follow-up examinations. Of those, 2334 returned after 5 years (75.8% of survivors), 1952 after 10 years (76.7% of survivors) and 1149 after 15 years (56.1% of survivors)[9]. Over the period between the 10- and 15-year visits, 364 had moved, 81 were admitted to a nursing home and were too frail to participate, 454 refused to return and 496 had died.

All baseline and follow-up visits of the BMES were approved by the Human Research Ethics Committees of the University of Sydney and the West Sydney Area Health Service, and were conducted adhering to the tenets of the Helsinki Declaration. Signed informed consent was obtained from all participants at each examination visit.

### Measures

A comprehensive interviewer-administered questionnaire was conducted detailing socio-demographic characteristics, past histories of angina, acute myocardial infarct, stroke, arthritis, hypertension, diabetes, the presence of a walking disability (the use of a cane, crutches, walking frame or wheelchair) and any hospital admissions (at least overnight) in the 12 month period prior to each visit. We also asked questions regarding educational qualifications and defined the highest educational qualification obtained by the participant (tertiary, secondary).

#### Cognitive Function

The MMSE was used to screen for possible cognitive impairment at the 5-, 10- and 15-year follow-up examinations. In this report, we used information from the modified version of MMSE, termed MMSE blind, excluding vision-related items and containing remaining items conserving the purpose of assessing memory, calculation and orientation[10–14]. Tasks of MMSE blind were added resulting in a maximum score of 22 (indicating the best level of cognitive function), instead of 30 as in the full version As there is no consensus as to how great a change in MMSE score is indicative of a clinically significant change, previous studies have suggested that a change of at least 2 to 4 points is considered clinically significant, with a 90% confidence level for it to be a meaningful decline in cognitive function[15;16] We therefore defined a decline of ≥3 MMSE blind scores between the 5-year and 10- or 15-year follow-up visits to indicate possible decline in cognitive function, over either 5 years (between the 5- and 10-year) or 10 years (between the 5- and 15-year).

#### Visual Impairment

Visual acuity (VA) was measured in both eyes at all examinations using a retro-illuminated logarithm of minimum angle of resolution (logMAR) chart (Vectorvision CSV-100TM, Vectorvision, Inc, Daytona, OH). Distance VA was measured at 244 cm for each eye with current spectacle prescription if worn, followed by pinhole acuity. Either a previous spectacle prescription or an auto-refraction (Humphrey automatic refractor, model 597, Humphrey-Zeiss, Germany) provided the baseline for a subjective refraction. Subjective refraction was performed using a modified Early Treatment Diabetic Retinopathy Study (ETDRS) protocol. VA was recorded as the number of letters read correctly in each eye (from 0–70). If no letters could be read from the chart at 244 cm, visual acuity was further assessed at 61 cm, recorded as either count fingers, hand movements, light perception, or no light perception. VA used in this report refers to the best-corrected acuity after subjective refraction. VI was defined as best-corrected VA <6/12 (39 letters or less read).

#### Hearing Impairment

Hearing was assessed at the 5-, 10- and 15-year follow-up examinations by means of pure-tone audiometry (PTA), performed by an audiologist in sound-proof booths using THD-39 (total dynamic distortion-39) earphones and Madsen OB822 audiometers (Madsen Electronics, Denmark). Hearing loss (HL) was defined as the pure-tone average of the audiometric hearing thresholds at 0.5, 1, 2 and 4 kilohertz (kHz) (PTA_0.5–4 kHz_) >40 decibels (dB)[5;17].

#### Dual Sensory Impairment

Dual sensory impairment (DSI) was defined as the co-presence of both VI and HL detected at the baseline visit of this report (the 5-year follow-up visit of the BMES cohort).

### Statistical Analysis

SAS 9.2 software (SAS Institute, Cary, NC) was used for statistical analyses. Logistic regression models (PROC LOGISTIC) were used to assess the associations between VI, HL and DSI with possible cognitive decline over 5 and 10 years. All measures, VI, HL and MMSE were assessed at the 5-year and later follow-up examinations. VI and HL assessed at the 5-year follow-up examinations were used as the study factors, and MMSE assessed subsequently over the follow-up period were used to assess the decline in MMSE over time (outcome factor). In the primary analyses, VI was defined as VA<6/12 in the worse eye, and HL as average PTA_0.5–4 kHz_ >40dB in the worse ear, to include unilateral and bilateral impairment cases. In supplementary analyses, VI was defined as VA<6/12 in the better eye, and HL as average PTA_0.5–4 kHz_ >40dB in the better ear, to include bilateral impairment cases only and hence more severe cases.

All reported longitudinal associations were after adjusting for baseline age and sex initially. Further adjustments for factors known to be associated with cognitive impairment[18–21], including the presence of walking disability, living arrangements (whether or not they live alone), home ownership, the highest educational qualification obtained (trade qualification or higher), baseline MMSE score, having ≥3 major co-morbidities (angina, acute myocardial infarct, arthritis, hypertension and diabetes), history of depressive symptoms and having hospital admissions in the 12 month period prior to baseline. A history of stroke is known to be associated with a reduced cognitive function[22–24], hence persons with a past history of stroke were excluded (n = 98). Odds ratios (OR) and 95% confidence intervals (CI) are presented.

## Results

Of the 3654 baseline participants of the BMES, 2334 were re-examined at the 5-year follow-up visit. Of these 2334, 1605 (68.8%) had completed the MMSE in at least 2 follow-up examinations and therefore were included in this report. Compared to participants who attended the 5-year follow-up, participants who were alive but did not attend the follow-up examinations were more likely to be current smokers, to have a history of diabetes, late age-related macular degeneration and VI. Participants who had died by the 5-year follow-up were more likely to have more chronic co-morbidities[9]. Table 1 shows the baseline characteristics of persons with VI, HL and DSI measured at the 5-year follow-up examination. Compared to participants with no sensory impairments, those with DSI were more likely to be older, to live alone, to have a walking disability, self-reported history of depressive symptoms, 3+ major co-morbidities, hospital admissions 12 months prior to baseline, and a lower mean MMSE blind score.

**Table 1:** Characteristics of Blue Mountains Eye Study participants with visual impairment, hearing loss and dual sensory impairment compared to participants with no sensory impairment measured at the 5-year follow-up examination.

Baseline characteristics	No Sensory impairment^a^ n (%)	Visual impairment^b^ n (%)	Hearing Impairment^c^ n (%)	Dual Sensory impairment^d^ n (%)
**N**	1308	152	330	93
**MMSE blind score**^e^	21.1 (1.5)	20.6 (2.1)	20.3 (2.3)	19.3 (2.8)
**Age (years)**^f^	66.9 (7.4)	74.3 (8.4)	73.4 (7.8)	80.4 (7.0)
**Gender (Female)**	544 (41.6)	52 (34.2)	171 (51.8)	40 (43.0)
**Lives alone**	303 (23.2)	60 (39.5)	94 (28.5)	42 (45.2)
**Walking disability**	62 (4.7)	24 (14.6)	48 (14.6)	34 (36.6)
**Home owner**	1228 (94.0)	136 (89.5)	303 (91.8)	83 (89.3)
**Hospital admissions 12 months prior**	306 (23.5)	43 (28.5)	78 (23.8)	33 (35.9)
**History of depressive symptoms**	129 (10.7)	15 (11.5)	40 (13.1)	13 (18.1)
**3+ major co-morbidities**	140 (10.7)	33 (21.7)	53 (16.2)	19 (20.9)

^a^ No visual or hearing impairment

^b^ Visual impairment defined as (Visual acuity <6/12) in the worse eye

^c^ Hearing loss defined as (>40 dB) in the worse ear

^d^ Visual and hearing impairment

^e^ Mini-mental state exam

^f^ mean (standard deviation)

Among 1352 participants who had MMSE assessed at both the 5- and 10-year follow-up examinations, possible cognitive decline was found in 9.5% (9), 11.1% (24) and 18.8% (6) of persons with VI, HL and DSI, respectively, compared to 7.6% of controls over the same 5-year period. Of 860 participants, who had MMSE assessed at both the 5- and 15-year follow-up examinations, possible cognitive decline was found in 21.9% (25), 20.8% (10) and 30.0% (3) of persons with VI, HL and DSI, respectively, compared to 16.0% of controls over a 10-year period.

Compared to participants with no sensory impairment, participants with VI, HL or DSI were not significantly associated with possible cognitive decline over 5 years (OR 0.84, 95% CI 0.40–1.79, OR 1.02, 95% CI 0.61–1.70 and 1.41, 95% CI 0.54–3.72, respectively), after adjusting for age and sex (Table 2). Similarly, participants with VI, HL or DSI were not significantly associated with possible cognitive decline over 10 years (OR 1.09, 95% CI 0.52–2.30, OR 1.09, 95% CI 0.65–1.82 and 1.15, 95% CI 0.28–4.73, respectively) (Table 3). Older age was found to be significantly associated with possible cognitive decline over both periods of 5 and 10 years, with an identical magnitude of association (per year increase in age, OR 1.07, 95% CI 1.04–1.10).

**Table 2:** Age- and sex-adjusted associations between sensory impairment and possible cognitive decline (≥3 units) in mini-mental state exam blind scores over 5 years.

	Decline ≥3 MMSE ^g^ blind scores		
Sensory impairment	n (%)	OR	95% CI
**Visual impairment**	9 (9.5)	0.84	0.40–1.79
**Hearing loss**	24 (11.1)	1.02	0.61–1.70
**Dual sensory impairment**	6 (18.8)	1.41	0.54–3.72

^g^ mini-mental state exam

**Table 3:** Age- and sex-adjusted associations between sensory impairment and possible cognitive decline (≥3 units) in mini-mental state exam blind scores over 10 years.

	Decline ≥3 MMSE ^g^ blind scores		
Sensory impairment	n (%)	OR	95% CI
**Visual impairment**	25 (21.9)	1.09	0.52–2.30
**Hearing loss**	10 (20.8)	1.09	0.65–1.82
**Dual sensory impairment**	3 (30.0)	1.15	0.28–4.73

^g^ mini-mental state exam

There were no significant changes in these associations between VI, HL or DSI and possible cognitive decline after further adjusting for other potential confounding variables: walking disability, living arrangements, home ownership, the highest educational qualification obtained, baseline MMSE score, a history of depressive symptoms, hospital admissions in the past 12 months and having 3 or more major chronic co-morbidities (data not shown).

Supplementary analysis using VI in the better eye and HL in the better ear showed no significant associations with possible cognitive decline over either 5-years (OR 1.19, 95% CI 0.64–2.20 for bilateral VI and OR 1.04, 95% CI 0.22–4.93 for bilateral HL, respectively), or 10-years (OR 1.35, 95% CI 0.72–2.53 for bilateral VI and OR 2.08, 95% CI 0.46–9.43 for bilateral HL, respectively). Bilateral DSI, defined using VI in the better eye and HL in the better ear, showed similarly no significant associations with possible cognitive decline over 5 years (OR 6.48, 95% CI 0.37–113.0). We could not assess the association between DSI and possible cognitive decline over 10 years due to small numbers.

## Discussion

In this older Australian cohort, we found that higher proportions of participants with visual, hearing or dual sensory impairment had a decline in 3+ MMSE blind scores after 5 and 10 years compared to participants with no sensory impairment. However, we did not find a significantly greater risk of possible cognitive decline after adjusting for age and sex, or further adjusting for more potential confounding variables. Older age was significantly associated with an increased risk of cognitive decline.

Very few longitudinal studies on the associations between sensory impairment and cognitive function have been reported[5;6], summarized in Table 4. The Study of Osteoporotic Fractures (SOF)[5] examined 6112 women age 69+ years and reported a cognitive decline by ≥3 scores approximately after an average of 4.4 years, using a modified MMSE. Using comparable definitions of sensory impairments to our study, SOF study investigators found that VI and combined vision and hearing impairment at baseline was associated with possible cognitive decline (OR 1.78 95% CI 1.21–2.61 and OR 2.19 95% CI 1.26–3.81, respectively), after adjusting for education, smoking, vertebral fracture, body mass index (BMI), grip strength, social network and baseline cognitive status[5]. However, the modified version of the MMSE (3MS) used in this study did not exclude vision-related items.

**Table 4:** Summary of the available evidence supporting the longitudinal associations between sensory impairment and cognitive function.

Author	Age (years)	Number	Follow-up Duration (years)	Cognitive function assessment tool & *change measures*	Adjusted variables	Association
Lin MY et al (2004) [5] SOF	69+	1333	Mean 4.4	-Modified MMSE (3MS), *Change ≥3 points*	Education, Smoking, Vertebral fracture, BMI, Grip strength, Social network	
*VI (BCVA<6/12)*						OR 1.78, 95% CI 1.21–2.61
*HI (40dB @2kHz)*						OR 2.38, 95% CI 0.95–2.00
*VI and HI*						OR 2.19 95% CI 1.26–3.81
Valentijian SAM et al (2005) [6] (MAAS)	55–83	418	6	-VVLT, SCWT, CST, LDST, *Parallel change in sensory function with cognitive function*.	Age, sex and education	
*Change in VA*, *Change in Hearing*						Parallel decline of both visual and hearing acuity with cognitive function using various cognitive function assessment tools
Reyes-Ortiz et al (2005) [7] (H-EPESE)	65+	2140	7	-MMSE-Blind: *Difference in the decline of average MMSE blind points per year*, *compared to persons without the corresponding impairments*	Age, sex, marital status, living arrangements, education, hypertension, stroke, diabetes mellitus, heart attack, depressive symptoms, and number of ADL limitations	
*Bilateral Near VI (<23-point type size)*						-0.62 SE±0.29
*Distance VI (<6/18)*						-0.06 SE±0.36
*HI (Hearing Hand-icap Inventory for the Elderly score <10)*						-0.04 SE±0.37
Lin FR et al (2013) [25] Health ABC, *HI PTA>25dB*	70–79	1162	6	-Modified MMSE (3MS) *Annual decline*	Age, sex, race, education, study site, smoking status, hypertension, diabetes and stroke history	-(-0.65 3MS points) -0.73—0.56
Gleniss V et al (1991) [26]	60–89	112	5	-JCST, WMS, Correlation *between a change in memory function and change in hearing*	Age and sex	
*Decline in Hearing*						Persons Correlation 0.3

BCVA—Best Corrected Visual Acuity

CST—Concept Shifting Task

JCST—Jacobs Cognitive Screening Test

HCT—Halstead Category Test

Health ABC—Health, Aging and Body Composition study

H-EPESE—Hispanic Established Populations for Epidemiological Studies of the Elderly

HI—Hearing Impairment MAAS—Maastricht Aging Study

LDST—Letter-Digit Substitution Test

MMSE—Mini Mental State Exam

SCWT—Stroop Colour Word Test

SE—Standard Error

SOF—Study of Osteoporotic Factures

WMS—Wechsler Memory Scale

VI—Visual Impairment

VVLT—Visual Verbal Learning Test

The Maastricht Aging Study [6] examined 418 participants aged 55+ years. The study investigators found that a deterioration of VA was in parallel with deterioration in cognitive function over a 6-year period. Various cognitive performance tests were used in this study, and although some tests were vision-related, the study investigators stated that the font size was large enough for all participants to perceive the words well. The finding of paralleled decline in both sensory and cognitive functions could suggest overall neurodegenerative changes associated with ageing.

The Hispanic Established Populations for Epidemiologic Studies of the Elderly[7] examined 2140 Mexican Americans aged 65+ years, and showed that participants with near VI (<7-point type size) at baseline had an average decline of 0.13 more MMSE-blind points per year than the decline in persons with normal near vision over 7 years (p = 0.045). They found no difference in per year change of MMSE-blind scores between persons with binocular distance VI and those with normal distant vision at baseline (p = 0.14)[7]. Vision assessment methods used in this study were not standardized; nevertheless, the latter finding from this study is consistent with our findings.

Longitudinal studies on the associations between hearing loss and cognitive function have also been inconsistent (Table 4). Both the Health, Aging and Body Composition study[25] (3075 participants aged 70–79 years) and the Maastricht Aging Study[6] (418 participants aged 55+ years), found that baseline hearing impairment was associated with a decline in cognitive function after 5 and 6 years, respectively. However, a study by Glennis et al[26] did not find any significant associations between hearing impairment and subsequent cognitive function after 5 years among 122 participants aged 60+ years. Different methods used to assess cognitive function across these studies make comparison with our findings difficult.

Our negative findings on the associations of interest may suggest that previously reported positive associations between sensory function impairment and reduced cognitive function testing scores are likely due to the reduced ability to process stimuli in persons with sensory impairment that compromised the performance on cognitive function testing[6;27].

Strengths of our study include its large population-based sample with reasonable follow-up rates and standardized methods to assess vision and hearing. Limitations include the relatively small number of participants with visual or hearing impairment who were followed. The MMSE is a screening tool, which should not be considered equivalent to specific diagnostic tools for cognitive impairment, and a decline of 3 MMSE score may not be a valid representation of cognitive decline despite this level of change has been used in previous studies[5]. We could not adjust for other unmeasured potential confounders. Additionally, although we adjusted for 3 or more major chronic co-morbidities as a potential confounding variable, each individual co-morbid condition does not have equal magnitude of association with cognitive decline. Furthermore, improvement in vision or hearing may have occurred subsequent to cataract surgery or the use of hearing aids during the course of the follow-up period which may have contributed to the lack of association between sensory impairment and cognitive decline. Another limitation is survival bias: persons who were unable or refused to attend the follow-up visits, or who had died, would be more likely to have been older and have had more co-morbid conditions including sensory impairment, and may have been more likely to experience cognitive decline.

In summary, we did not find significant associations between sensory impairment and subsequent decline in MMSE scores after adjusting for age and sex, or after additional adjustment for multiple potential confounders. The use of MMSE blind may not be an adequate screening tool to detect a decline in cognitive function. Alternatively, a reduced cognitive function measure may be related to a reduced ability to process stimuli, both of which concurrently exist among persons with sensory impairment but their correlation is consistent overtime. Longitudinal studies with larger sample sizes, clearly defined sensory impairment duration, and the use of appropriate testing tools to disentangle cognitive impairment from sensory impairments, are needed to confirm or refute the hypothesis that sensory impairment may lead to an increased risk of cognitive decline among older persons.

## Supporting Information

S1 Dataset Complete supporting dataset. (XLSX)
